# The Application and Microstructural Analyses of Newly Developed Luster Glazes on Different Clay Bodies

**DOI:** 10.3390/ma19163405

**Published:** 2026-08-11

**Authors:** Merve Öztorun, Nermin Demirkol

**Affiliations:** 1 Department of Handicrafts, Program of Ceramic and Glass Design, Technical Sciences Vocational School, Istanbul University-Cerrahpaşa, Istanbul 34500, Türkiye; merve.oztorun@iuc.edu.tr; 2 Department of Ceramics, Faculty of Fine Arts, Kocaeli University, İzmit 41300, Türkiye

**Keywords:** luster glaze, microstructure (SEM-EDS), white clay, chamotte clay, silver nitrate

## Abstract

This study describes the microstructural and chemical characteristics of luster glazes applied to white clay and chamotte clay bodies using scanning electron microscopy coupled with energy-dispersive X-ray spectroscopy (SEM–EDS) and X-ray diffraction (XRD). Surface and cross-sectional analyses were performed to evaluate glaze morphology, the glaze–body interface, and the formation of metallic phases responsible for the luster effect. SEM observations indicated that the glaze applied to the white clay body exhibited greater thickness uniformity and a well-defined transition zone. In contrast, the chamotte clay body, due to its higher porosity, showed a more heterogeneous glaze distribution and increased interfacial irregularities. EDS analyses revealed the localized distribution of metallic elements, particularly silver and copper, and demonstrated the influence of body composition on glaze–body interactions. XRD results confirmed the presence of silver- and copper-rich crystalline phases on the glaze surface. Overall, the results demonstrate that the optical and esthetic performance of luster glazes depends on both glaze formulation and the microstructural properties of the ceramic body, providing relevant insights for optimizing artistic and industrial applications.

## 1. Introduction

Luster glazes represent a traditional ceramic surface decoration technique that imparts an optically rich, metallic, and iridescent appearance to ceramic surfaces through the formation of thin metallic layers. The luster technique was first developed in the 8th century CE during the Abbasid Caliphate, when glass artisans in Egypt applied metallic compounds onto transparent glass surfaces under controlled firing conditions [1,2,3]. This innovation later extended to ceramic production and became a distinctive feature of Islamic ceramic art.

The earliest luster-glazed ceramics date to the 9th century CE and were produced during the Abbasid period in Mesopotamia, particularly in regions surrounding the caliphal palace in Samarra, as well as in major ceramic centers such as Basra, Kufa, and Baghdad. Additional early examples have been identified in Fustat, near Cairo, associated with the Fatimid dynasty. Lusterware production subsequently expanded across the Islamic world, with notable examples from Syria dated between the 12th and 15th centuries CE and from Iran between the 12th and 17th centuries CE. The technique was later transmitted to North Africa and introduced to Spain, from where it spread into Europe [4]. Archeological findings in Málaga, including luster-glazed bowls embedded in church walls and dated to the mid-13th century CE, attest to this cultural and technological transmission.

From a technical perspective, luster formation involves the reduction of metal compounds, leading to the development of a thin, pearlescent metallic film on the glaze surface [5,6,7]. This film consists of a glassy matrix containing nanoscale silver and copper particles or ions embedded within the surface layer, which are responsible for the characteristic metallic reflections and optical effects [1,8]. Depending on the kiln atmosphere, luster techniques can be classified into reducing and oxidizing types. Reducing atmosphere lusters include in-glaze and paste lusters, while oxidizing atmosphere lusters comprise resonant, vaporization, and spray lusters [9,10].

In contemporary ceramic practice, although the visual outcomes of luster applications may differ from historical examples, the fundamental physicochemical mechanisms remain largely unchanged. The success of the luster effect is governed not only by glaze formulation and firing parameters but also by the interaction between the glaze layer and the underlying ceramic body. Understanding these interactions at the microstructural and chemical levels is therefore essential for optimizing luster glaze performance in both artistic and industrial ceramic applications.

Recent studies have significantly improved the understanding of luster glaze formation. Pradell et al. demonstrated that metallic luster develops through an ion-exchange mechanism followed by the reduction of silver and copper species, leading to the formation of nanostructured metallic layers within the glaze surface [1,6]. Subsequent studies further revealed that glaze composition, firing conditions, and the development of Ag/Cu nanoparticles strongly influence the optical properties of luster glazes [7]. However, relatively few studies have investigated the influence of ceramic body characteristics, particularly body microstructure and glaze–body interactions, on luster formation.

SEM–EDS and XRD have been widely employed to investigate the microstructure, elemental distribution, and crystalline phases of historical and contemporary luster glazes, providing valuable information on nanoparticle formation and glaze–body interactions [5,6]. However, these techniques have rarely been applied to comparatively evaluate the influence of different ceramic body microstructures on luster development.

Considering the limited number of studies on the influence of ceramic body characteristics, this study comparatively investigates the behavior of newly developed luster glazes applied to white clay and chamotte clay bodies. The microstructure, glaze thickness, integrity of the glaze layer, and glaze–body interfacial interactions were examined using scanning electron microscopy (SEM) and energy-dispersive X-ray spectroscopy (EDS). In addition, X-ray Diffraction (XRD) analysis was performed to characterize the metallic phases present on the glaze surface. The results provide further insight into the influence of ceramic body microstructure on luster glaze formation and may contribute to the optimization of luster glazes for both artistic and industrial ceramic applications.

## 2. Materials and Methods

### 2.1. Development and Calculation of Ceramic Glazes

A lead-free boron–alkali glaze was formulated using Hermann Seger’s calculation method. When fired at 1000 °C in an oxidizing atmosphere, the glaze produced a glossy, transparent surface [11]. The raw materials, obtained in milled form from the company Refsan Industrial Furnace and Machine Company., Kütahya, Türkiye are listed in Table 1. Glaze preparation and firing were performed at the Ceramic Technology Laboratory, Faculty of Fine Arts, Kocaeli University.

The alkali and boron-containing recipe developed as part of Oztorun and Demirkol’s study was used in this study [12]. Using the base recipe developed in this study, 5 new glaze recipes were created using different metal oxides and metal salts (Table 2).

For the experimental study, the raw materials were weighed using a precision balance in accordance with the proportions specified in Table 1. The glaze batch was then prepared by wet milling with the addition of water in a ball mill for 30 min to achieve a homogeneous mixture. The milling process was carried out using a Double-Chamber Mill 2200 (stock code: R6002) supplied by Refsan Industrial Furnace and Machine Company, Kütahya, Turkey. Each experimental glaze composition was prepared by precisely weighing 100 g of the glaze suspension. The metal oxides listed in Table 2 were incorporated into the glaze formulations at the specified proportions. The samples prepared by adding the following to the base batch recipe shown in Table 1 are as follows: Sample A: 3 wt. % CoO, Sample B: 2 wt. % CoO, 3 wt. % CuO, Sample C: 3 wt. % Fe_2_O_3_, 1 wt. % CuO and Sample D: 10 wt. % CuO, 1 wt. % Cr_2_O_3_, 0,5 wt. % SnO_2_ (Table 2).

Silver nitrate (AgNO_3_) was added as a metal salt at a concentration of 2 wt. % relative to the total glaze weight. Following the addition of the metal compounds, the glaze suspensions were mixed in a ball mill for 30 min to ensure compositional homogeneity.

The prepared luster glazes were evenly brushed onto the surfaces of the ceramic test specimens. The glaze suspensions were applied manually using a soft brush in two successive coats while the specimens were held horizontally. The second coat was applied after the first coat became touch-dry to obtain a uniform glaze layer over the entire surface. After application, the coated samples were dried at room temperature prior to firing.

### 2.2. Sample Preparation and Firing Process

Specimens of white and chamotte clay bodies were fabricated by pressing plastic clay into plaster molds. During firing, clay minerals undergo dehydroxylation, particle rearrangement, and sintering, resulting in microstructural and mineralogical changes that influence the final properties of the ceramic body [13]. The samples were biscuit fired in an electric kiln at 1000 °C. Four different glaze recipes were formulated by adding various metal oxides and silver nitrate to the base mix. The same glazes were applied to both the white and chamotte bodies using a brush.

First, the prepared recipe was applied with a brush to a sample obtained from a white body, and oxidation firing was carried out in an electric kiln. After firing, the sample exhibited transparent, shiny properties. Subsequently, the recipe was modified by adding the metal oxide and silver nitrate shown in Table 2, and it was applied with a brush to both the white body and biscuit bodies obtained from chamotte clay. Reduction firing was then performed in a gas-fired kiln.

**Figure 1:**
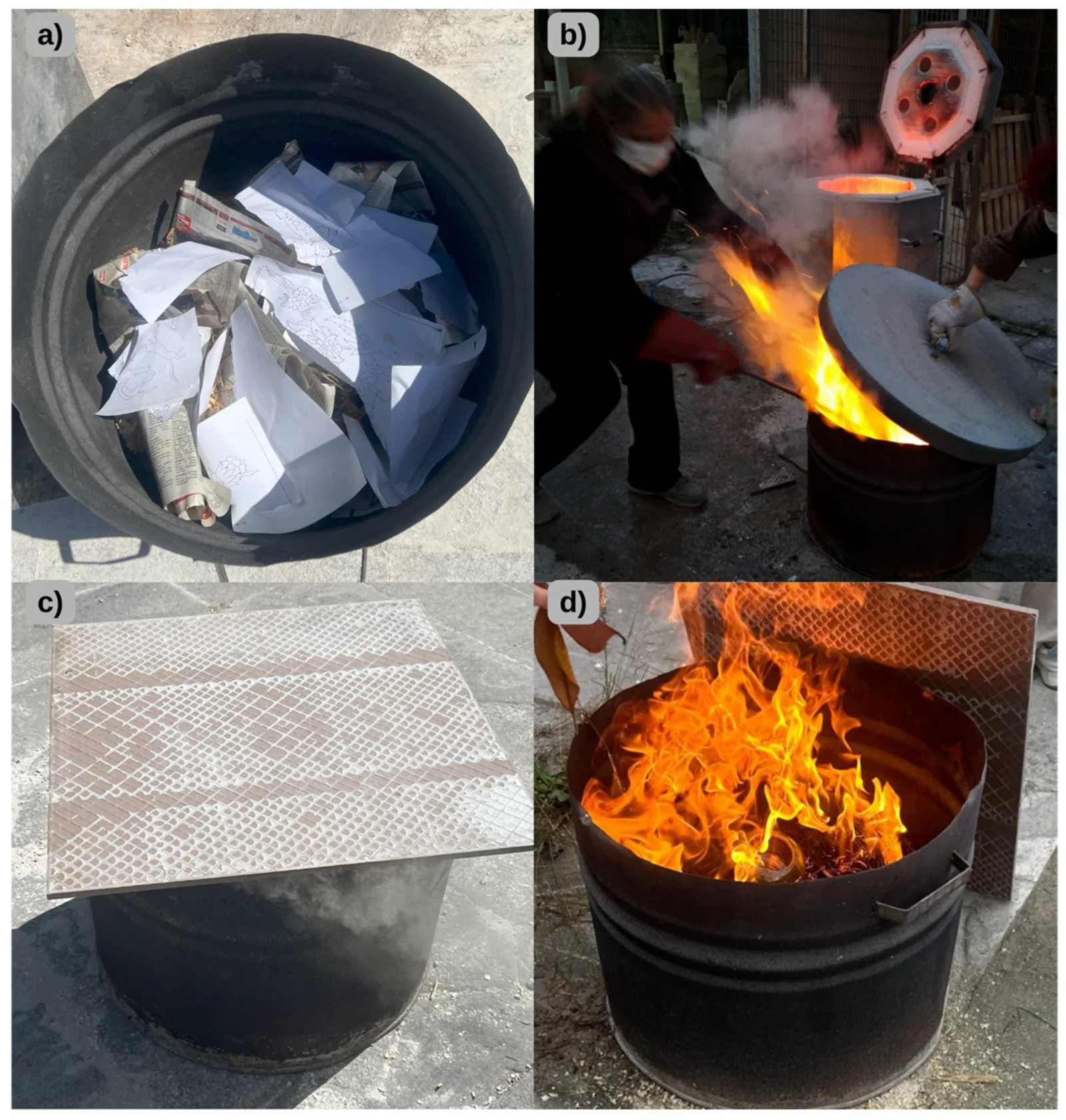
For the reduction process of the samples: (**a**) organic waste such as shavings, newspaper, dried fruit, and nut shells prepared in advance in a metal barrel, (**b**) transferring the samples taken from the furnace into the metal barrel, (**c**) closing the lid of the metal barrel containing the samples and leaving them for a 20 min reduction process, (**d**) opening the lid of the metal drum containing the samples to complete the reduction process.

After glazing, the samples were fired in a gas kiln using bottled gas. The kiln was programmed to reach 1000 °C within a total firing duration of 2 h. The temperature was held at 1000 °C for 10 min, after which the gas supply was shut off and the kiln was allowed to cool to 850 °C. To create a reducing atmosphere, a metal barrel was preloaded with wood shavings, newspaper, walnut shells, hazelnut shells, and onion skins. At 850 °C, the kiln door was opened, and the samples were transferred into the metal barrel, where they were subjected to reduction for 20 min (Figure 1) [12].

After the 20 min reduction process, the samples were removed from the metal barrel and allowed to cool to room temperature. Following visual examination, the samples were characterized by SEM–EDS and XRD analyses.

### 2.3. Microstructural and Phase Analyses

The microstructural characteristics of the reduction-fired luster-glazed samples were investigated using scanning electron microscopy (SEM) (JEOL Ltd., Tokyo, Japan) coupled with energy-dispersive X-ray spectroscopy (EDS). Analyses were performed with a JEOL JSM-6060VL microscope (JEOL Ltd., Tokyo, Japan). Prior to examination, the samples were gold-coated for 90 s to ensure surface conductivity. SEM imaging was conducted at an accelerating voltage of 15 kV and 100× magnification, and elemental compositions were determined from the same regions using the integrated EDS system.

Phase analysis was carried out using a Rigaku Ultima III X-ray diffractometer (Rigaku Ultima III, Tokyo, Japan) operated at 40 kV and 40 mA with CuKα radiation (λ = 0.154 nm). Diffraction patterns were collected over a 2θ range of 5–80° at a scanning rate of 2° min^−1^. The glazed surfaces of the fired specimens were directly analyzed without additional surface treatment.

Microstructural characterization analyses were performed using SEM, EDS, and XRD analyses conducted with equipment available at the Department of Metallurgy and Materials Engineering at Sakarya University.

## 3. Results

### 3.1. Visual Appearance of the Luster Glazes

The results obtained from the samples after reduction firing were quite successful. In addition to the metallic effect of gold, silver, and copper, a rainbow luster effect is clearly visible. The decomposition of sulfates and nitrates creates an atmosphere inside the furnace, and this atmosphere reduces copper and silver compounds by one mole at temperatures between 500 °C and 600 °C. Cu^2+^ is reduced to Cu^+^ [14,15]. Ion exchange occurs between silver and copper ions (Ag^+^, Cu^2+^, and Cu^+^) and the alkali ions (Na^+^ and K^+^) present in the glaze, and the reduction of Ag^+^ to Ag^0^ and Cu^2+^/Cu^+^ to Cu^0^ results in the growth of metal nanoparticles [16].

In general, oxidation-reduction conditions depend on the amount of metal salts and metal oxides present in the recipe. Therefore, the reduction atmospheres used in the furnace will vary for each recipe [17,18]. Green-yellow gold glazes are obtained using compounds containing silver, while red ruby and red copper glazes are obtained using compounds containing copper [15]. Yellow, brown, dark brown, amber, and orange, non-metallic effects are obtained using compounds containing both copper and silver. Although bright layers were obtained across the entire temperature range studied between 450 °C and 600 °C, the most suitable effects were obtained at a temperature of 550 °C [19].

**Figure 2:**
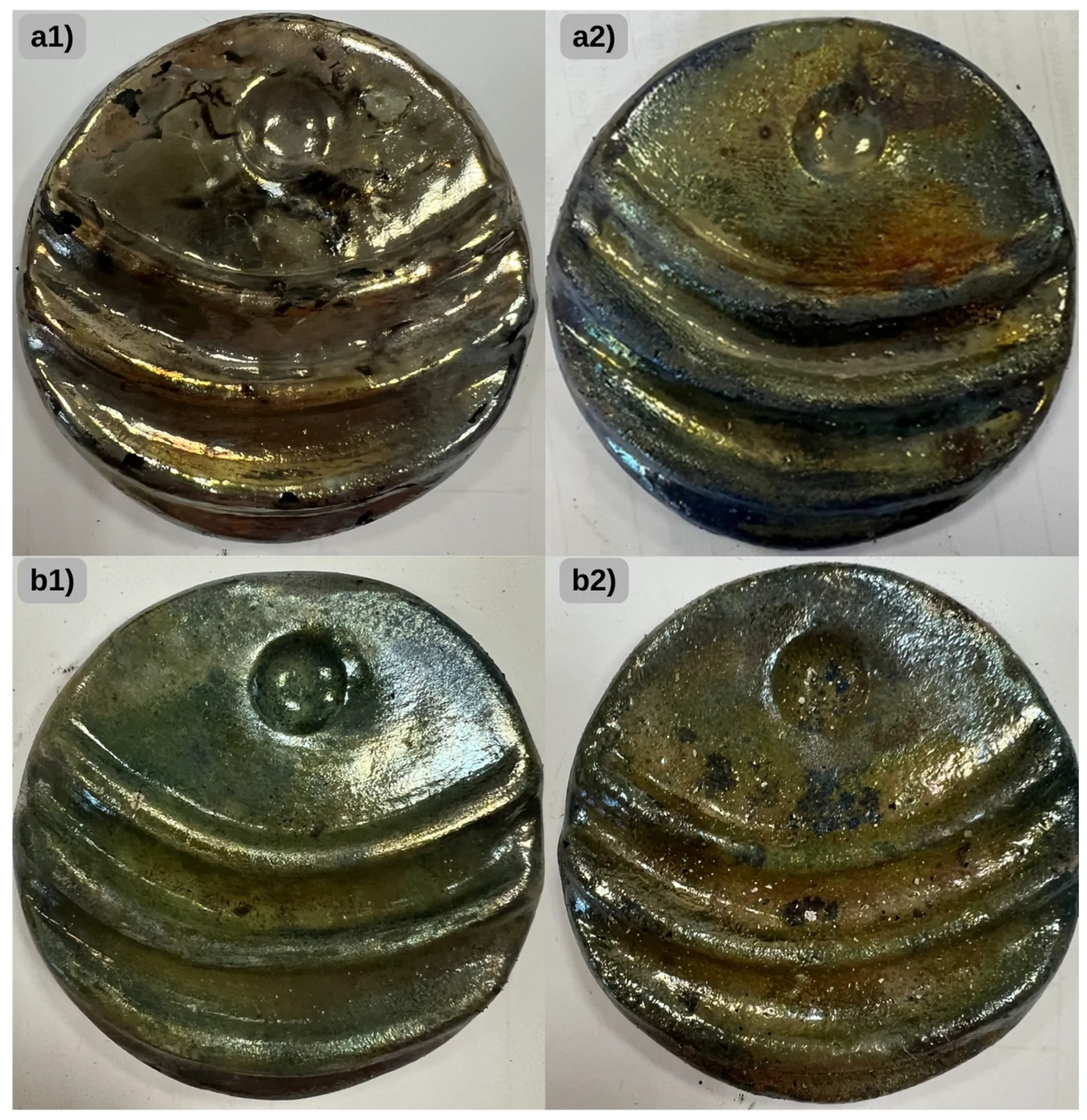
After the reduction process, the results for Samples A: (**a1**) white body, (**a2**) chamotte body and, Samples B: (**b1**) white body, (**b2**) chamotte body.

**Figure 3:**
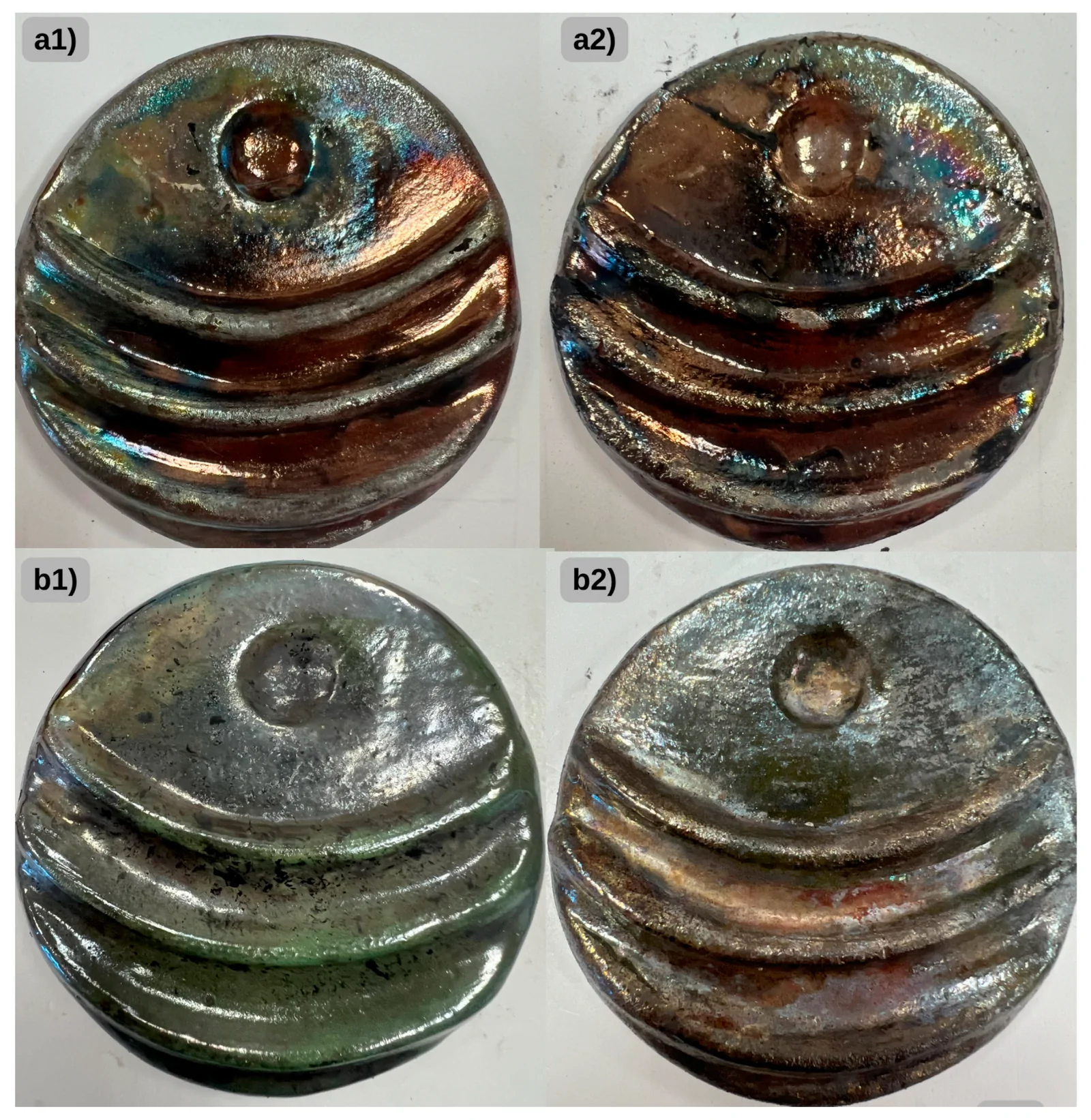
After the reduction process, the results for Samples C: (**a1**) white body, (**a2**) chamotte body and Samples D: (**b1**) white body, (**b2**) chamotte body.

In addition to the metallic effect, reddish tones from CuO after reduction, the brown tones from Fe_2_O_3_, the blue tones from CoO, and the green tones from Cr_2_O_3_ are also visible. The results of the reduction cooking are shown in Figure 2 and Figure 3.

Samples A1, B1, C1, and D1 correspond to the white body, while Samples A2, B2, C2, and D2 represent the chamotte body after reduction firing, as shown in Figure 2 and Figure 3 respectively.

The golden-yellow reflections observed in the visual analyses of Samples A1 and A2 are attributed to the reduction of 2 wt. % silver nitrate (AgNO_3_) added to the glaze recipe into metallic silver nanoparticles (Ag^0^) under a reducing atmosphere. The diffusion coefficient of silver ions within the glassy matrix is directly correlated with the alkali content and boron concentration of the glaze. High concentrations of alkali ions (Na^+^, K^+^) facilitate the migration of silver ions toward the surface, leading to the characteristic luster sheen through the surface plasmon resonance (SPR) effect [20]. The deep blue tones observed in the samples are associated with the dissolution of 3 wt. % cobalt oxide (CoO) in the borosilicate matrix in the Co^2+^ ionic state (Figure 2).

Samples B1 and B2 contain a combination of 2 wt. % CoO, 3 wt. % CuO, and 2 wt. % AgNO_3_ (Table 2). In the luster glaze applied to the white body (Figure 2(b1)), reduction firing promoted the migration of copper oxide and silver nitrate toward the glaze surface, where they were reduced to form a thin metallic film layer. This resulted in green–gold hues and, in certain regions, iridescence-like metallic reflections. The reduction of silver nitrate to metallic silver enhanced surface brightness and light reflectivity, while cobalt oxide, despite its lower concentration, contributed to increased color depth by darkening green tones and introducing cooler optical reflections within the luster layer.

In contrast, the glaze applied to the chamotte body (Figure 2(b2)) exhibited a more irregular luster effect characterized by speckled and locally matte regions. The high porosity of the chamotte body led to partial absorption of the glaze and metal ions into the body, thereby limiting the continuity of the metallic film formed at the surface. The reduction of copper oxide to Cu^0^/Cu_2_O phases under reducing conditions resulted in dark green, brownish, and locally near-black surface features. These effects are attributed to localized accumulation of metal ions and non-uniform reduction across the surface.

In both bodies, reduction firing played a decisive role in the formation of the metallic luster layer. However, on the white body, the more controlled accumulation of metal ions at the surface produced a more homogeneous and brighter luster. Conversely, the surface roughness and porosity of the chamotte body caused diffuse light scattering, yielding a more textural, random, and natural luster esthetic.

Samples C1 and C2 contained 3 wt. % Fe_2_O_3_, 1 wt. % CuO, and 2 wt. % AgNO_3_ in their glaze composition. On the white body, the glaze exhibited a metallic copper and iridescent appearance, with silver reflections observed in places (Figure 3(a1)), while on the chamotte body, brown copper, gold, and silver reflections were observed (Figure 3(a2)). The luster formation mechanism produced very similar results in the white and chamotte bodies. Only the glaze layer thinned due to the porous structure of the chamotte body, and a decrease in the luster effect was observed compared to the white body. For luster formation, not only the glaze composition but also the absorption capacity of the ceramic body and the thickness of the glaze layer are very important [21,22].

Samples D1 and D2 contained 10 wt. % CuO, 1 wt. % Cr_2_O_3_, 0.5 wt. % SnO_2_, 1 wt. % CuSO_4_, and 2 wt. % AgNO_3_. On the white body (Figure 3(b1)), green background tones with localized silver metallic reflections were observed, whereas on the chamotte body (Figure 3(b2)), in addition to green hues, reddish copper and silver reflections were evident. In the white body, incompletely reduced copper ions (Cu^2+^) remained trapped within the glaze matrix, producing the characteristic green coloration, while silver nanoparticles accumulated at the surface as a gray/metallic layer without generating sufficient optical interference in thicker glaze regions [1].

In the chamotte body, the more efficient penetration of reducing gases (CO) into the glaze depth facilitated the transformation of copper ions into metallic copper (Cu^0^) or colloidal red copper oxide (Cu_2_O), resulting in reddish coloration. In the presence of chromium oxide (Cr_2_O_3_) and tin oxide (SnO_2_), the metallic nanoparticles within the thin glaze layer enhanced light scattering and exhibited higher luster brightness. The absorptive behavior of the chamotte body dynamically modified glaze viscosity during firing, enabling the formation of a homogeneous and continuous thin metallic film at the surface [22].

### 3.2. Scanning Electron Microscopy (SEM) Analyses

**Figure 4:**
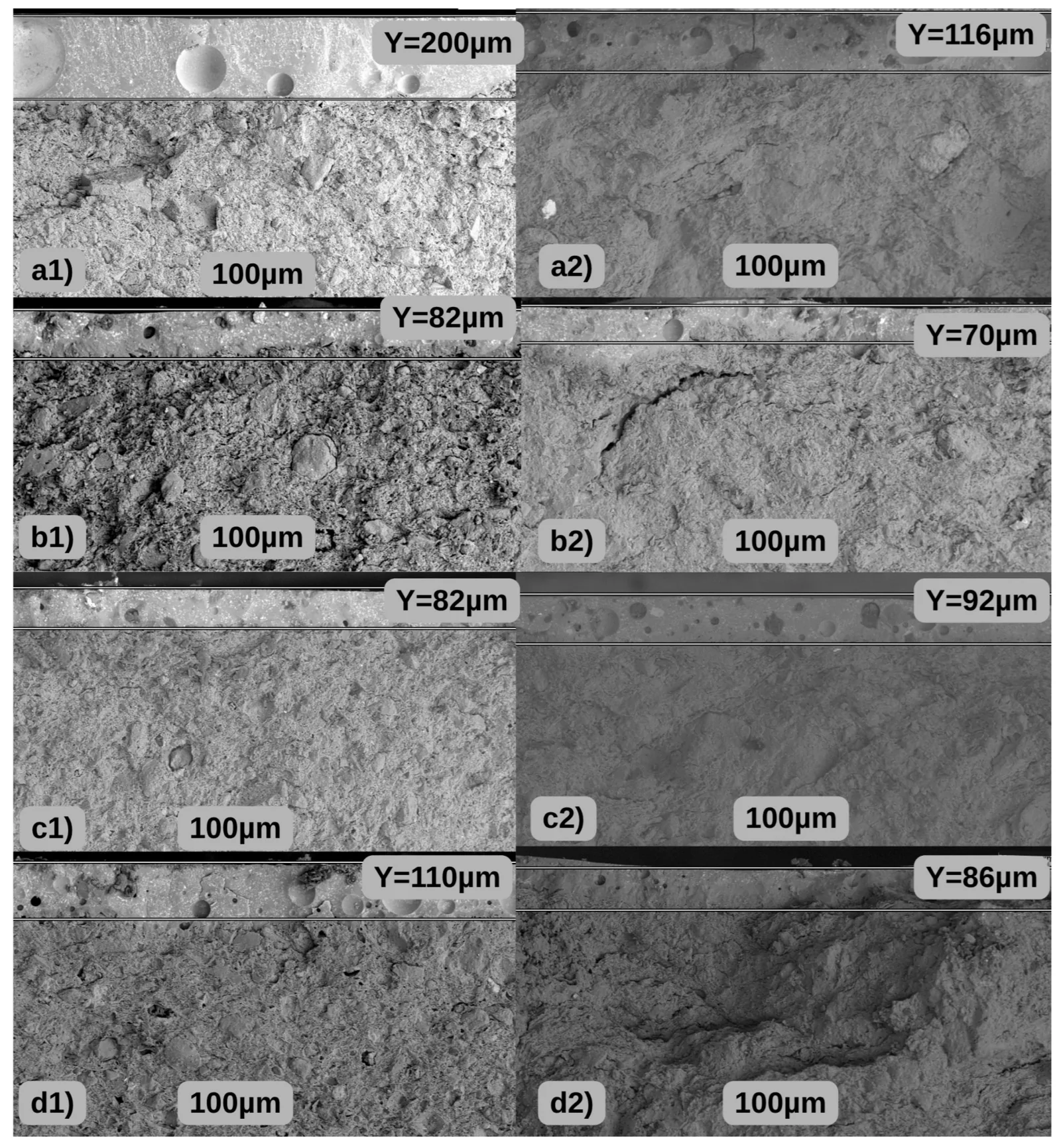
Cross-sectional SEM micrographs showing the glaze thickness and glaze–body interface of luster-glazed samples. (**a1**–**d1**) White clay body and (**a2**–**d2**) chamotte clay body corresponding to glaze formulations A–D.

Cross-sectional micrographs were obtained using a scanning electron microscope operated in backscattered electron (BSE) mode. In the grayscale SEM images, the darker regions correspond to pore spaces, whereas the lighter regions represent the ceramic particles, allowing the evaluation of pore distribution and microstructural characteristics [23,24]. Representative cross-sectional SEM micrographs showing the glaze thicknesses of the luster glazes applied to white clay and chamotte clay bodies after reduction firing are presented in Figure 4.

**Figure 5:**
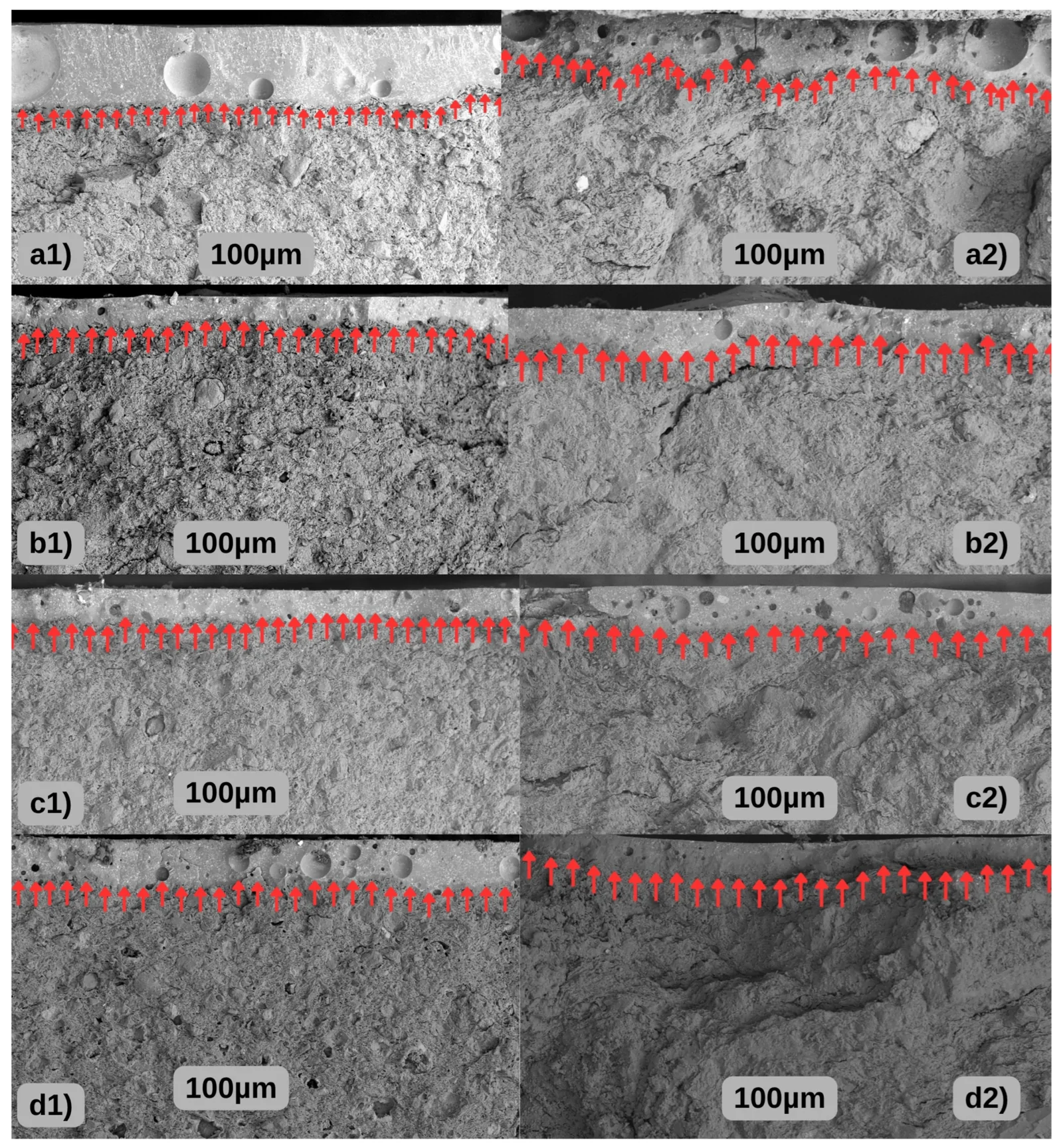
SEM cross-sectional images showing the glaze–body interfaces of samples (**a1**–**d2**), as indicated by red arrows.

Figure 5(a1–d1) show that the interface between the white clay body and the glaze is more uniformly distributed. It was observed that the interface between the chamotte clay body and the glaze exhibits a more irregular distribution (Figure 5(a2–d2)).

The chamotte body exhibits a more porous and heterogeneous structure due to the presence of fired ceramic particles (chamotte). Air trapped within the voids between chamotte grains tends to expand during firing, promoting the formation of larger gas bubbles in the glaze layer. The macro-bubbles observed in the SEM images reflect the aggressive nature of the chamotte body during dehydration and gas release stages. However, the micro-rough surface generated by chamotte particles can hinder the homogeneous distribution of silver nanoparticles. In contrast, gas release in the white clay body is more controlled; bubbles are smaller and more uniformly distributed, allowing the luster film to remain continuous.

The influence of body composition on luster development becomes particularly evident when comparing chamotte and white clay samples. The high porosity and surface roughness of the chamotte body can scatter the surface plasmon resonance (SPR) effect of silver nanoparticles, thereby diminishing metallic brightness [1]. Moreover, trace amounts of iron oxide diffusing from the chamotte body into the glaze matrix may shift the golden coloration produced by silver toward darker or dirtier tones. The white clay body, being free from such contamination, enables the chromophoric oxides and the golden-yellow color derived from silver nitrate to develop in their purest and highest saturation state [22]. The glaze penetrates more deeply into the body, resulting in stronger mechanical interlocking. Because the white body is finer-grained and more homogeneous, it forms a smoother glaze–body interface, which promotes more uniform light reflection (specular reflection) and enhances the brightness of the luster effect [25,26].

Additionally, analyses of the chamotte body reveal greater variability in alumina (Al_2_O_3_) content at the glaze–body interface compared to the white clay body. This variation locally alters glaze viscosity, resulting in a characteristic mottled appearance of the luster.

The glaze exhibits a generally homogeneous vitreous phase with the presence of circular microporosities. This microstructural feature is attributed to volatile species released during the decomposition of raw materials such as ulexite and borax, which became trapped due to the relatively high viscosity of the glaze melt [27].

The continuity of the glaze–body interface indicates good compatibility between the thermal expansion coefficients of the glaze and the ceramic body, confirming successful fusion. The base glaze composition used in the samples (Table 1), characterized by high boron content (20 wt. % ulexite and 20 wt. % borax) and alkali enrichment, provides a suitable matrix for low-temperature luster development.

Figure 4(a1) presents the SEM cross-sectional image of Sample A glaze applied on the white body. In Sample A1, the glaze layer thickness was measured to be approximately 200 µm (Figure 4). Figure 4(a2) shows the SEM cross-sectional image of Sample A glaze applied on the chamotte body. In Sample A2, the glaze layer thickness was measured to be approximately 116 nm, and the glaze–body interface exhibits a heterogeneous distribution.

SEM cross-sectional images of the glossy glaze applied to white body sample B (Figure 4(b1)) reveal a homogeneous and continuous glaze layer 82 µm thick. The glaze-body interface exhibited a more heterogeneous distribution compared to sample A. It is anticipated that this situation may be due to the glaze being applied unevenly and thinly onto the body.

SEM image of body sample B containing chamotte (Figure 4(b2)) shows a heterogeneous glaze thickness of 70 µm. The presence of coarse chamotte particles results in a rough and irregular glaze-body interface. In several areas, partial glaze penetration into the porous body is evident, resulting in localized thinning of the glaze layer. This type of microstructural heterogeneity is consistent with previous studies indicating that highly porous or grog-containing bodies disrupt the formation of a uniform glaze layer and negatively affect luster development [21].

Both samples were fired in a reducing atmosphere after adding 2% CoO, 3% CuO, and 2% AgNO_3_ by weight to the glaze formulation. SEM observations revealed the formation of a very thin metallic film layer near the glaze surface in both cases. The reason for the lack of luster on the surface is that the Cu and Ag remain embedded within the glaze matrix. However, while the white-body glaze appears more uniform, the chamotte-body glaze exhibits localized agglomerations. This difference can be explained by the stronger adsorption of metal ions onto the porous chamotte body, resulting in reduced accumulation of these ions on the glaze surface during reduction, where metallic nanoparticle formation occurs [20].

Microstructural examinations reveal that the glaze thickness on the white body (Figure 4(c1)) is 82 µm, while that on the grogged (fireclay) body (Figure 4(c2)) is 92 µm. The glaze thicknesses on the white body and the chamotte body have formed an ideal glaze layer for luster formation. The glaze layers observed on both bodies have enabled Ag ions to migrate to the surface, ensuring a homogeneous thermal distribution and complete maturation, resulting in a consistent metallic film capable of constructive optical interference [12,21,27].

The gray–silver luster and green background observed on the white body (Figure 4(d1)) can be attributed to the incomplete reduction of copper in the lower regions of the 110 µm-thick glaze layer. The low porosity of the white body, combined with the relatively thick glaze layer, effectively kept the system below the optimal melting point or hindered gas evacuation due to high glaze viscosity. As a result, silver (Ag) accumulated irregularly at the surface, producing a gray appearance, while copper in the underlying layers retained its green chromatic character [11].

In contrast, a glaze thickness of 86 µm on a chamotte body represents a critical threshold for luster formation. The open porosity provided by the chamotte (grog) particles not only resulted in a thinner and more irregular glaze layer but also facilitated the faster penetration of reducing gases from the kiln atmosphere into deeper regions of the glaze (Figure 5(d2)). Copper was more densely concentrated in the layers near the surface of the chamotte sample and, by localizing within the glaze matrix, led to the emergence of vivid red and lustrous silver and copper-like colors [21] (Figure 3(b2)).

SEM images have revealed that the glaze thickness on the chamotte bodies decreases due to glaze diffusion. However, it should not be forgotten that another possible cause of these variations in glaze thickness could stem from differences in the application of the glaze to the body using a brush.

### 3.3. Energy-Dispersive X-Ray Spectroscopy (EDS) Analyses

In the regional EDS analysis of the glaze layer, Si originates from quartz, feldspars, and kaolin; Al from feldspars and kaolin; Na from borax and sodium feldspar; Ca from ulexite and dolomite; Mg from dolomite; and Zn from the ZnO additive.

EDS analyses acquired from the marked points on the SEM images confirm that the glaze layer is primarily composed of Si and O, forming a silicate-based glass matrix. Moreover, the EDS spectra collected from surface-near regions exhibit pronounced characteristic peaks corresponding to Ag, indicating that silver ions migrated toward the glaze surface during reduction firing and were reduced to metallic Ag^0^ [22]. The higher intensity of Ag peaks detected particularly in near-surface analysis points suggests that silver plays a dominant role in the formation of a thin metallic film, which is the primary source of the perceived gold and bronze coloration.

The coexistence of Ag signals with peaks associated with glaze constituents such as Na, Al, and Si in the EDS spectra indicates that the metallic silver phase is either dispersed within the glass matrix or precipitated as nanometer-scale structures in surface-adjacent regions [23]. These findings support the correlation between the microstructural heterogeneity observed in SEM images and the optically observed metallic and iridescent effects, which are strongly associated with the enrichment of metal ions such as Ag and Cu in near-surface regions of the glaze layer [21].

Ag nanoparticles were detected via EDS point analysis from the shadow of the coating layer near the surface in the 50 µm SEM image of Sample A. The Cu and Ag present in Sample B produced close peaks at a single EDS point. The high concentration of CuO in Sample C produced a very high peak at a single EDS point, while Ag, being proportionally much less than CuO, corresponded to a low peak. In Sample D, point analysis of the glaze layer from the 50 µm SEM image revealed a Si peak, and other elements present in small amounts in the glaze were also detected.

**Figure 6:**
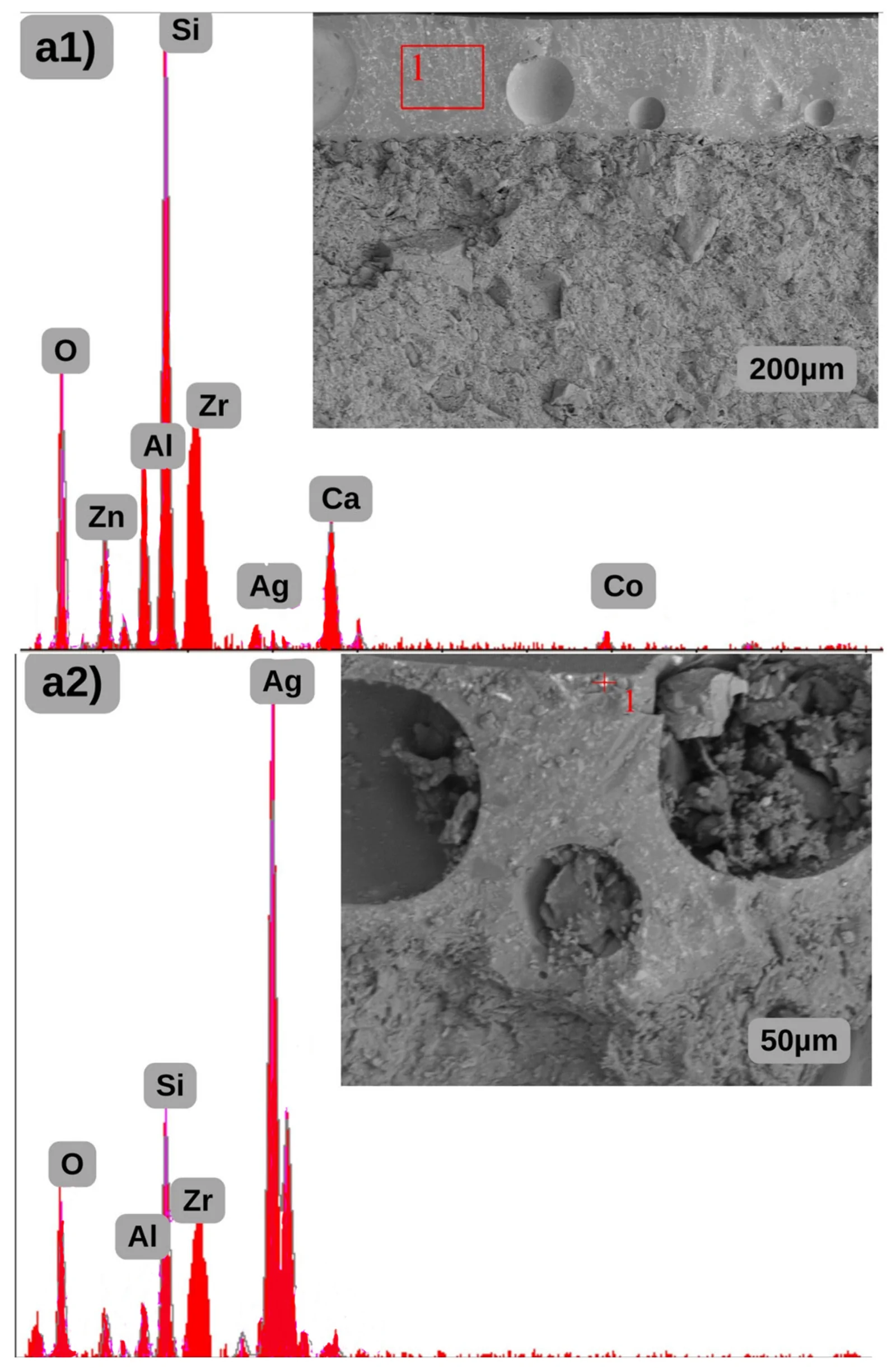
SEM cross-sections and EDS analyses of Sample A glaze on white (**a1**) and chamotte (**a2**) bodies, showing Ag enrichment near the surface and a more heterogeneous glaze–body interface in the chamotte body.

The ionic exchange between Na+ or K+ and Cu+ and Ag+ in the solution occurs during firing in a reducing atmosphere. During this reduction phase, nanocrystals of metallic copper and metallic silver are formed. The typical sizes of the nanocrystals are 15 to 20 nm for copper Cu and 5 to 20 nm for silver Ag. The composition of the glaze is very important for the development of the luster film layer [20]. Figure 6(a1) and Figure 6(a2) present the SEM cross-sectional images of the samples coated with Sample A glaze applied to the white body and chamotte body, respectively. The rectangular area indicated in Figure 6(a1) and the marked point (+1) in Figure 6(a2) correspond to the regions selected for EDS analysis. The point EDS analysis confirms that silver forms localized concentrations in the near-surface regions of the glaze layer (Figure 6(a2)), whereas the area EDS analysis verifies that cobalt exhibits a stable and homogeneous distribution throughout the glaze matrix (Figure 6(a1)).

The addition of zirconium oxide (ZrO_2_) introduces a slight opacity beneath the luster layer, enhancing the contrast of the reflected light and reinforcing the optical depth of the surface [28].

EDS spectra collected from the glaze surface regions of both samples confirm the presence of major glaze-forming elements, including O, Si, Al, Na, K, and Ca, originating from feldspar, borate sources, and clay components [29]. In addition, metal elements responsible for the luster effect were clearly detected.

**Figure 7:**
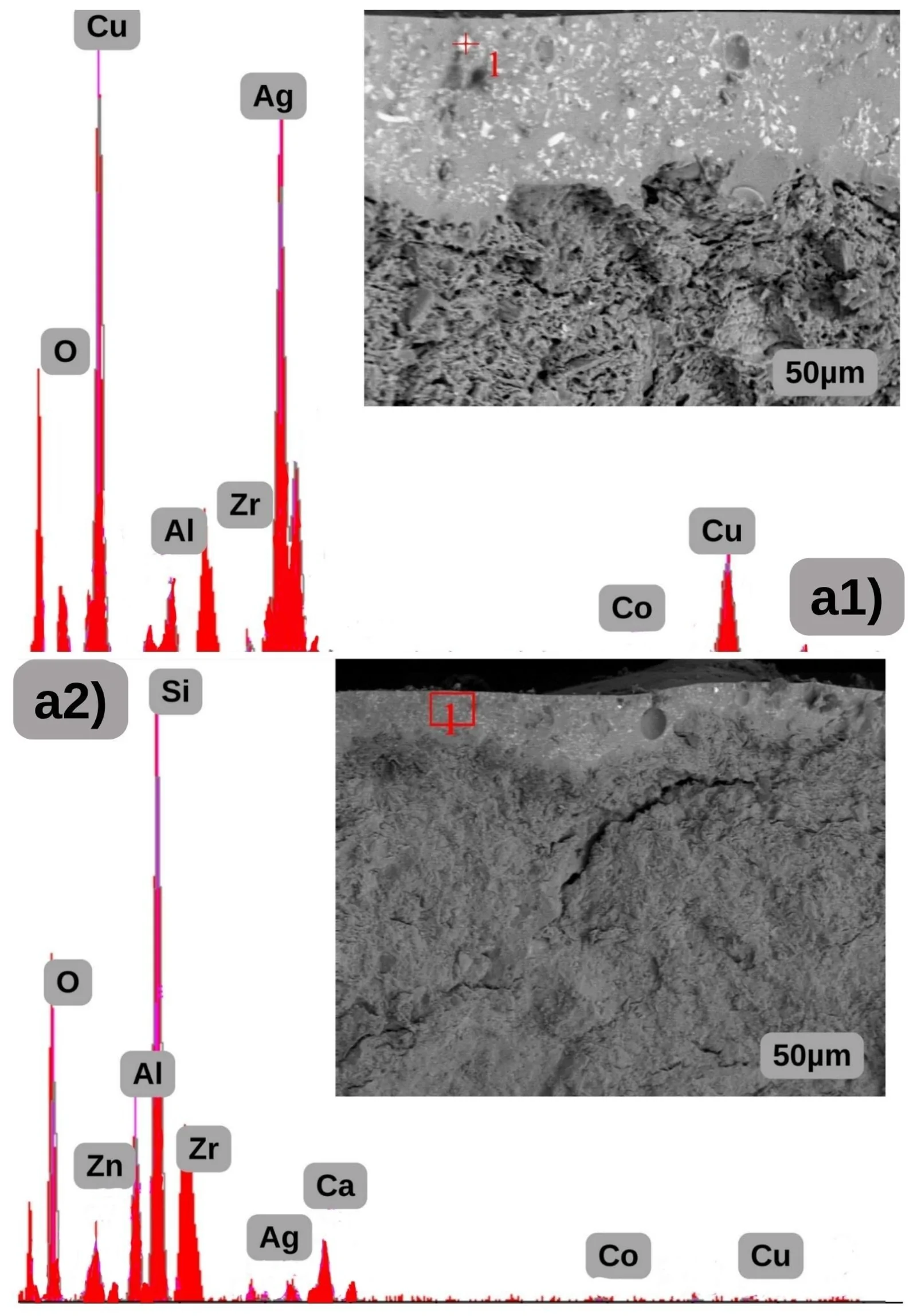
SEM cross-sections and EDS analyses of Sample B glaze on white (**a1**) and chamotte (**a2**) bodies, showing Ag enrichment near the surface and a more heterogeneous glaze–body interface in the chamotte body.

For the white body sample (Figure 7(a1)), EDS analyses show pronounced Ag and Cu peaks, particularly in surface-near regions, indicating the accumulation and reduction of silver ions into metallic silver nanoparticles [30]. The presence of Cu and Co peaks further supports their role in modifying the color tone and contributing to the optical depth of the luster layer [29]. The relatively sharp and intense Ag peaks suggest effective metal ion migration toward the glaze surface under reduction conditions, which is consistent with classical luster formation mechanisms [20].

In the chamotte body sample (Figure 7(a2)), Ag, Cu, and Co peaks are also detected; however, their intensities are comparatively lower and more broadly distributed. This observation indicates partial diffusion of metal ions into the porous body structure rather than surface concentration [31]. Additionally, stronger Al and Si peaks are observed due to the contribution of chamotte particles, which dominate the EDS signal in localized regions and reflect the heterogeneous microstructure of the body–glaze interface [32].

**Figure 8:**
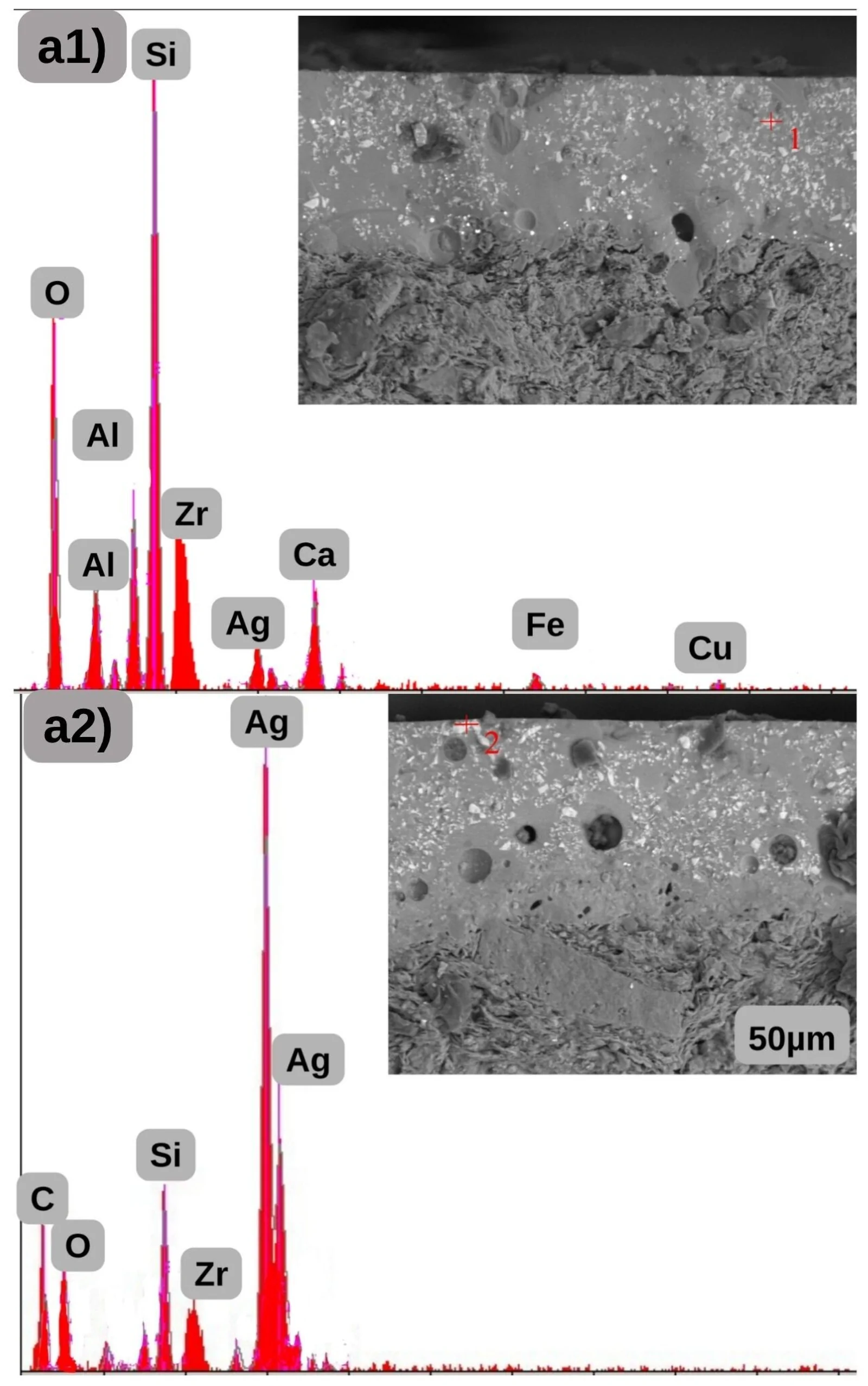
SEM cross-sections and EDS analyses of Sample C glaze on white (**a1**) and chamotte (**a2**) bodies, showing Ag enrichment near the surface and a more heterogeneous glaze–body interface in the chamotte body.

The point EDS analysis (+1) identified in the SEM cross-sectional image of Sample C white clay body was taken from regions deeper than the surface of the glaze layer. This analysis confirmed the elemental composition of the raw materials present in the glaze’s general formulation (Figure 8(a1)). The spectrum obtained revealed the presence of basic glass-forming and auxiliary oxides belonging to the glaze matrix, and no significant metallic phase concentration was detected (Figure 8(a2)) [11,28,31,33].

**Figure 9:**
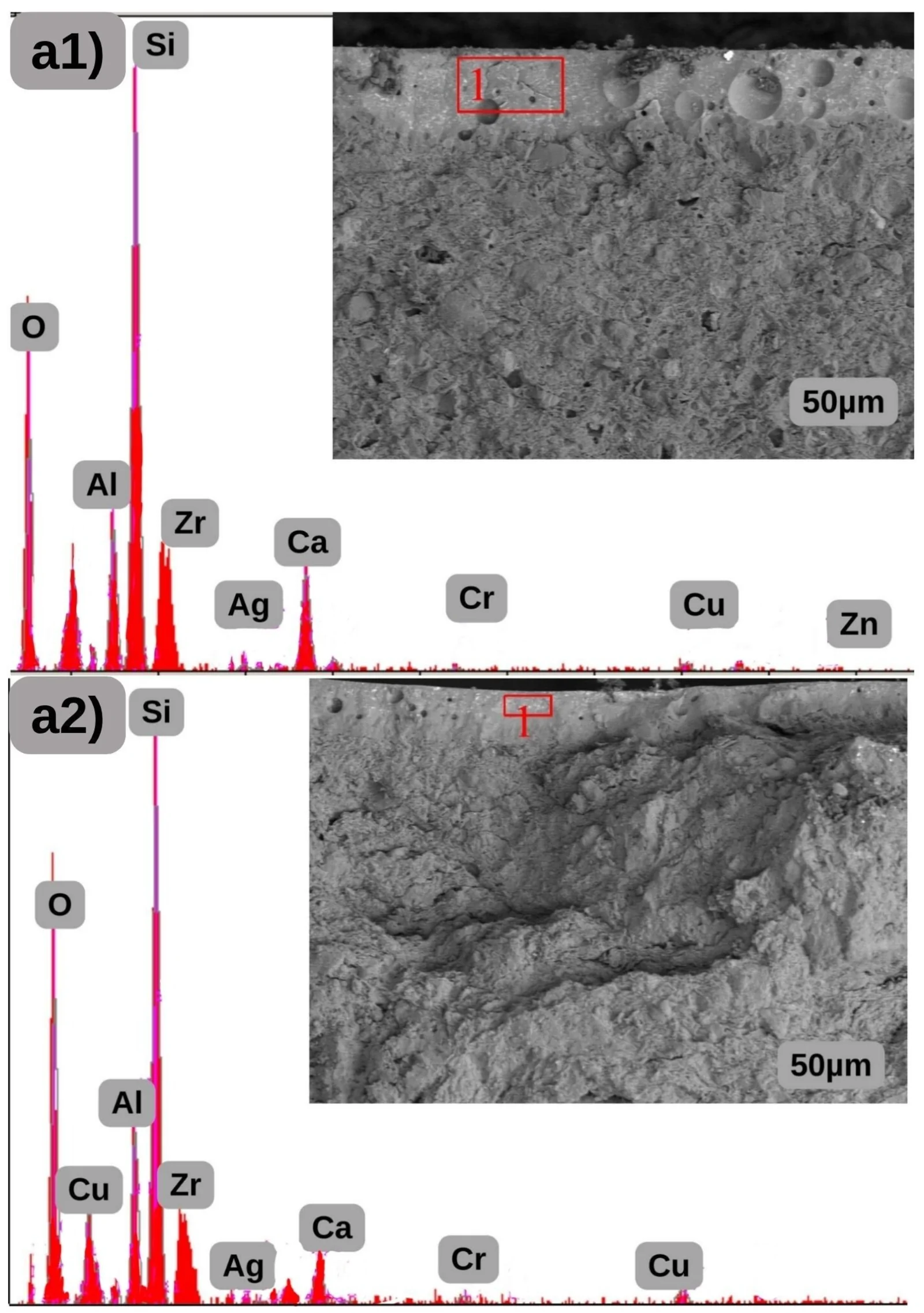
SEM cross-sections and EDS analyses of Sample D glaze on white (**a1**) and chamotte (**a2**) bodies, showing Ag enrichment near the surface and a more heterogeneous glaze–body interface in the chamotte body.

The optical performance of luster glazes depends not only on the chemical composition of the glaze but also on the microstructural characteristics of the ceramic body and the resulting elemental distribution profile. Area EDS analyses conducted on the white body revealed high oxygen (O) and silicon (Si) contents, indicating that copper (Cu) and silver (Ag) are diluted within the glassy matrix rather than forming a continuous metallic film on the surface (Figure 9(a1,a2)). These observations are consistent with incomplete reduction of copper species reported in previous studies [1]. Nevertheless, localized effects of reduction can still be observed in certain regions.

### 3.4. X-Ray Diffraction (XRD) Analyses

**Figure 10:**
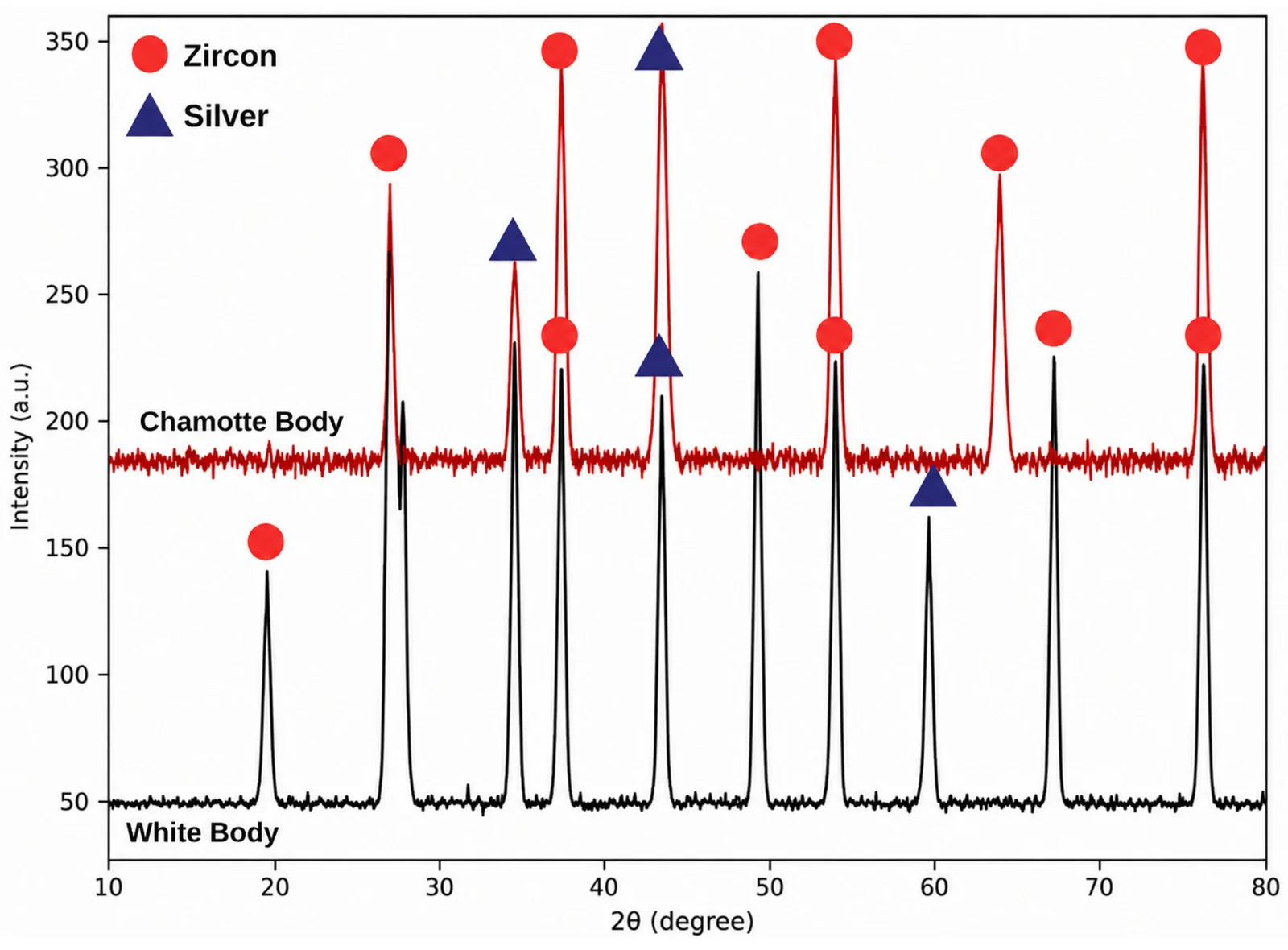
Surface XRD analyses of the chamotte bodies of sample C.

The surface XRD phase analyses of Sample C in the white body and chamotte body are shown comparatively (Figure 10). It reveals that the main crystalline phases formed under reducing firing conditions are zircon (ZrSiO_4_) and metallic silver (Ag) [20,32].

The zircon phase is quite dominant in the samples. It has been observed that zircon increases the opacity effect on the surface rather than the luster effect. In contrast, Ag reflections are moderate, indicating controlled silver reduction and finely dispersed metallic crystallites within or near the glassy matrix, which is related to the observed homogeneous gold luster and strong specular reflection. The Ag ions responsible for the formation of the metallic luster film have been confirmed by surface phase analysis [6,34]. Overall, the results indicate that the quality of brilliance is determined not by the maximum crystalline silver content but by the microstructural balance and distribution of the metallic phase within the glassy matrix.

### 3.5. Overall Interpretation of the Microstructural Findings

The combined SEM–EDS and XRD analyses indicate that luster formation is influenced by both glaze composition and ceramic body characteristics. The white body exhibited a more homogeneous glaze layer, whereas the higher porosity of the chamotte body promoted more effective reduction and metallic phase development. Variations in glaze thickness and glaze–body interactions affected the distribution of Ag and Cu species, resulting in differences in luster appearance. These findings are consistent with previous SEM studies demonstrating that pore distribution and particle arrangement are important microstructural parameters governing the behavior of clay-based ceramic materials [35]. Overall, the results demonstrate that body microstructure plays an important role in determining the final characteristics of reduction-fired luster glazes.

## 4. Discussion and Conclusions

In this study, the optical behavior of luster glazes was comprehensively evaluated in relation to glaze chemistry, metal oxide additions, and, most critically, the microstructural characteristics of the ceramic body. SEM cross-sectional observations and EDS analyses clearly demonstrate that luster formation is governed not only by the presence of metal ions but also by their distribution within the glaze–body system, their reduction behavior, and the continuity of the metallic film formed near the surface.

The white body, characterized by low porosity and a homogeneous microstructure, provided a smoother glaze surface; however, in thicker glaze layers (≈100 µm), copper ions (Cu^2+^) were not fully reduced. Area EDS analyses showing high O and Si contents indicate that copper and silver were diluted within the glassy matrix rather than forming a continuous metallic film. This microchemical configuration resulted in an absorption-based green background accompanied by an irregular gray–silver luster appearance. Although localized surface accumulation of silver ions was observed, insufficient thin-film homogeneity limited the development of strong interference-based luster brightness.

In contrast, the chamotte body, with its high open porosity, micro-rough surface, and thinner glaze layers (≈86 µm), enabled more efficient penetration of reducing gases into the glaze. SEM–EDS results revealed co-enrichment of copper and silver in near-surface regions, leading to the formation of a synergistic metallic film. This configuration promoted the interaction of Cu^0^/Cu_2_O and Ag^0^ nanoparticles, resulting in red, gold, and highly reflective metallic luster effects.

EDS analyses further identified the presence of impurities such as Fe_2_O_3_ and TiO in the chamotte body. While these elements locally suppressed metallic brightness through increased optical absorption, they also contributed to a more textured, heterogeneous, and natural luster esthetic. Conversely, the impurity-free white body allowed colorant oxides and silver-derived golden hues to appear with higher chromatic purity and saturation. It has been demonstrated that the difference in coating thickness affects ion mobility and luster development.

In conclusion, the performance of luster glazes cannot be attributed solely to glaze formulation. Instead, body porosity, microstructure, impurity content, glaze thickness, and the ability of the reducing atmosphere to penetrate the glaze layer should be considered as interrelated parameters governing luster formation and optical performance.

In this study, the bright transparent glaze formulation developed was successfully transformed into a luster glaze through the incorporation of metal oxides and metal salts under reducing firing conditions. During the reduction process, the ceramic specimens were removed from the kiln at approximately 850 °C and exposed to rapid cooling, creating severe thermal stresses within the ceramic body. Under these experimental conditions, the chamotte body exhibited satisfactory luster formation despite greater glaze diffusion associated with its higher porosity, indicating its suitability for reduction-fired luster applications where thermal shock resistance is an important consideration.

Although this study provides valuable insight into the influence of ceramic body microstructure on luster glaze formation, it is limited to two ceramic body types and the glaze formulations investigated under a single reduction-firing schedule. Future studies should examine a wider range of ceramic bodies, glaze compositions, and firing conditions to further clarify the relationships between body characteristics, glaze–body interactions, and the optical performance of luster glazes.

## Figures and Tables

**Table 1:** Semi-transparent glossy glaze recipe that can be fired at 1000 °C [12].

Raw Materials	(wt. %)
Ulexite	20
Borax	20
Potassium Feldspar	20
Sodium Feldspar	15
Zinc Oxide	2.5
Zircon Oxide	2.5
Dolomite	5
Kaoline	10
Quartz	5
Total	100

**Table 2:** Recipes for samples created with metal oxides and metal salts in a semi-transparent glaze.

Compositions Codes	Semi-Transparent Glaze (wt. %)	Metal Oxides (wt. %)	Metal Salt (wt. %)
Sample A	100	%3 CoO	%2 AgNO_3_
Sample B	100	%2 CoO + %3 CuO	%2 AgNO_3_
Sample C	100	%3 Fe_2_O_3_ + %1 CuO	%2 AgNO_3_
Sample D	100	%10 CuO + %1 Cr_2_O_3_ + %0.5 SnO_2_	%1 CuSO_4_ %2 AgNO_3_

## Data Availability

The original contributions presented in this study are included in the article. Further inquiries can be directed to the corresponding author.
